# Supplementary material for: The interactome of intact mitochondria by cross-linking mass spectrometry provides evidence for coexisting respiratory supercomplexes
Source: Mol Cell Proteomics. 2017 Dec 8;17(2):216–32. doi: 10.1074/mcp.RA117.000470 (PMC5795388; doi:10.1074/mcp.RA117.000470)
Supplement: Supplemental Data [file supp_RA117.000470_133922_0_supp_23978_fzffwf.zip › spectra_annotation/mito_DR_spectra_annotation/268-1-12-1-9-1.pdf]

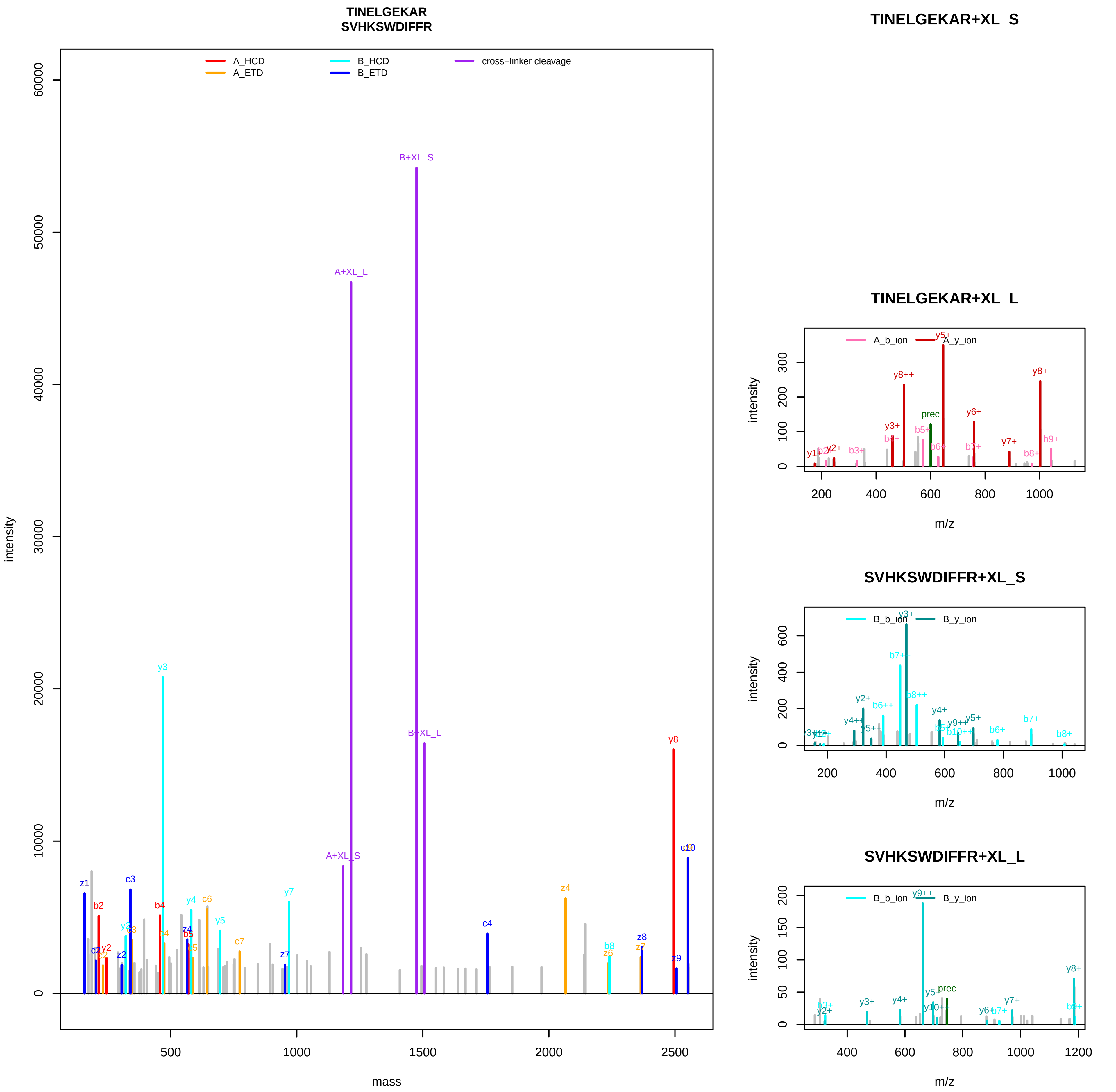
TINELGEKAR
SVHKSWDIFFR
A_HCD
A_ETD
B_HCD
B_ETD
cross−linker cleavage
B+XL_S
A+XL_L
B+XL_L
A+XL_S
y3
y8
c10
z1
b2
c3
b4
y4
c6
y5
c7
y7
c4
z4
b8
z6
z8
z7
z9
intensity
mass
0
10000
20000
30000
40000
50000
60000
500
1000
1500
2000
2500
TINELGEKAR+XL_S
TINELGEKAR+XL_L
A_b_ion
A_y_ion
y5+
y8++
y8+
y6+
prec
y3+
b5+
y7+
b9+
b8+
b3+
y2+
y1+
m/z
SVHKSWDIFFR+XL_S
B_b_ion
B_y_ion
y3+
b7++
b8++
y2+
b6++
y4+
y5+
b7+
b6+
b8+
m/z
SVHKSWDIFFR+XL_L
B_b_ion
B_y_ion
y9++
prec
y8+
y3+
y4+
y7+
m/z
intensity
